# Immunogenicity of a Recombinant Zoster Vaccine (gE/BFA01) in Mice

**DOI:** 10.3390/v18010053

**Published:** 2025-12-30

**Authors:** Yaru Quan, Shiqiang Luo, Shuang Wu, Kaiqin Wang, Lixing Hu, Yihuan Hao, Kangwei Xu, Yong Liu

**Affiliations:** 1 State Key Laboratory of Drug Regulatory Science (SKLDRS), National Institutes for Food and Drug Control, Beijing 102629, China; 2 Beijing ABZYMO Biotechnology Co., Ltd., Beijing 100176, China; 3 Jiangsu Recbio Technology Co., Ltd., Taizhou 225316, China

**Keywords:** varicella zoster virus, novel adjuvant, recombinant zoster vaccine, cellular immune responses, humoral immune responses

## Abstract

Varicella-zoster virus (VZV) is a human neurotropic herpesvirus. The primary infection with VZV causes chickenpox and establishes latency in sensory and dorsal root ganglia. Viral reactivation leads to herpes zoster (HZ), which is accompanied by complications such as postherpetic neuralgia (PHN), causing a significant disease burden. At present, vaccination is the most effective preventive measure. We developed a recombinant zoster vaccine, gE/BFA01, which comprises truncated VZV glycoprotein E and the liposome-based adjuvant BFA01 (containing MPL and QS-21). In this study, we evaluated the recombinant zoster vaccine’s immunogenicity in a live attenuated VZV-primed C57BL/6N mouse model and explored the mechanism of action of the BFA01 adjuvant. The results indicate that the gE/BFA01 vaccine induces superior antibody responses and stronger cellular immune responses compared with gE with aluminum hydroxide. Furthermore, gE/BFA01 showed comparable immunogenicity to the licensed vaccine Shingrix. Mechanistic investigations revealed that the BFA01 adjuvant can enhance the recruitment of innate immune cells at the injection site, increase the expression of DCs surface maturation markers, and activate multiple inflammatory signaling pathways in lymph nodes. Collectively, these findings indicate that gE/BFA01 can induce potent humoral and cellular responses, supporting its further development as a high-efficiency vaccine candidate.

## 1. Introduction

Shingles (herpes zoster (HZ)) is caused by the reactivation of the varicella-zoster virus (VZV), which remains latent in the dorsal root ganglia of the spinal cord, the sensory ganglia of the cranial nerves, and the trigeminal nerve following initial infection [1]. VZV has neurotropic and lymphotropic properties and can cause chickenpox in children following primary infection and shingles in adults following reactivation of latent infection [2,3]. Common complications of HZ include postherpetic neuralgia (PHN), neurological disorders, and ocular diseases. Among these, PHN is the most common and severe complication, with pain that can persist for months or even years, significantly impacting the patients’ quality of life [4,5,6,7]. The cumulative incidence rate of HZ in different countries and regions around the world is approximately 2.9–19.5 per 1000 person-years [8]. The incidence rate of HZ among the elderly population in China is approximately 10 per 1000 person-years [9,10]. A research model predicted that the current lifetime incidence risk of HZ is 32.4% and that of PHN is 2.8%; by 2035, the lifetime incidence risks of HZ and PHN will be 34.8% and 3.3%, respectively, representing increases of 7.5% and 16.8% compared with current levels [11].

Currently, there are no effective treatments for HZ, especially for PHN [12]. Vaccination against herpes zoster is an effective means of preventing HZ and PHN. There are two types of herpes zoster vaccines available on the market: live attenuated and recombinant protein vaccines [13]. Live attenuated vaccines have been shown to vary greatly in their protective efficacy between different age groups and have poor immunological persistence. Zostavax^®^, manufactured by Merck, has protection rates of 69.8% and 51.3% for people aged 50–59 and ≥60, respectively, while the protection rate drops to 37.6% for people aged 70 and above [4,14,15,16]. It has a certain preventive effect on PHN (66.5%), with the protection efficacy dropping to about 30% after six years [17]. Shingrix^®^, developed by GSK, is a recombinant zoster vaccine based on the gE protein and AS01_B_ adjuvant. It offers significant advantages in preventing HZ and PHN. The vaccine provides a 97.2% protection rate against HZ in individuals aged 50 or older and a 91.3% protection rate in those aged 70 or older [18,19]. It effectively reduces the risk of PHN (88.8%) and provides protection for over 10 years [20].

VZV gE is the most abundant type I transmembrane glycoprotein on the surface of VZV-infected cells [21], playing a crucial role in viral replication and intercellular transmission [22,23]. VZV gE contains epitopes recognized by B and CD4 T cells, and multiple VZV gE-based vaccines can induce high levels of antibody and cellular immune responses [24,25,26,27]. MPL (3-O-desacyl-4’-monophosphoryl lipid A) is derived from cell wall lipopolysaccharide (LPS), a ligand for the TLR4 receptor and its coreceptor MD2. Compared with LPS, MPL exhibits significantly reduced toxicity while retaining its pro-inflammatory effects [28]. TLR4-mediated activation of innate immune cells induces an inflammatory response that promotes Th1 differentiation and Th1-biased humoral immunity [29]. QS-21 is a saponin adjuvant extracted from the bark of the *Quillaja saponaria* tree from South America [30]. It has been demonstrated to influence antigen presentation to antigen-presenting cells (APCs) and promote the induction of cytotoxic T lymphocytes in animal models [31]. QS-21 undergoes cholesterol-dependent endocytosis and accumulates in endosomes. Lysosomal destabilization and cathepsin B activation are key processes in the QS-21-mediated activation of moDC responses [32]. Following endocytosis, QS-21 also mediates the formation of lipid bodies within dendritic cells [33], inducing the cross-presentation of antigens via PERK-dependent pathways [34].

Our team developed the recombinant zoster vaccine containing the gE antigen, as well as the BFA01 adjuvant, which contains two immunostimulatory molecules: MPL and QS21 [24]. This study evaluated the immunogenicity of gE/BFA01 in VZV-primed female C57BL/6N mice, simulated a clinical setting where most individuals at higher risk for herpes zoster are VZV seropositive [35,36,37,38], and conducted immunogenicity comparisons with the marketed recombinant herpes zoster vaccine Shingrix, providing data support for subsequent clinical studies of this vaccine. To further investigate the action of mechanism of the BFA01 adjuvant, we conducted experiments on innate immune cell recruitment and lymph node dendritic cell (DC) activation, and performed lymph node transcriptome analysis studies targeting the BFA01 adjuvant.

## 2. Materials and Methods

### 2.1. Recombinant gE and Adjuvant

Recombinant zoster vaccine contains truncated varicella-zoster virus glycoprotein E (gE protein) and a BFA01 adjuvant, with specification of 50 μg/0.5 mL/vial. The coding sequence of VZV gE was truncated to remove the transmembrane anchoring domain and C-terminal domain. The VZV gE protein was expressed in Chinese hamster ovary cells and then purified. The purified gE protein was analyzed using SDS–PAGE and SEC-HPLC (Supplementary Figure S1A,B), the purity detected using SEC-HPLC was 96.7%. The BFA01 adjuvant is a liposome-based adjuvant that contains MPL (100 μg/mL) and QS21 (100 μg/mL) and is developed by Jiangsu Recbio Technology Co., Ltd. (Taizhou, China). The particle size of the BFA01 adjuvant is about 100 nm (Supplementary Figure S1C).

The Alhydrogel™ adjuvant (Al(OH)_3_) was purchased from Croda International PLC. HZ/su (Shingrix^®^, GlaxoSmithKline Biologicals SA, London, Brentford, UK) consists of 50 μg of recombinant gE antigen and AS01_B_ adjuvant.

### 2.2. Vaccine Immunogenicity

#### 2.2.1. Mouse Immunization

The 6–8-week-old specific-pathogen-free (SPF) female C57BL/6N mice used in this experiment were purchased from Beijing Vital River Laboratory Animal Technology Co., Ltd. (Beijing, China). All mice were housed with free access to water and food under SPF conditions. All mice were maintained in regular day and night light cycles (12 h light/12 h dark) in a barrier facility located in the AAALAC-accredited Beijing Yizhuang International Biomedical Technology Co., Ltd. (Beijing, China).

**Figure 1:**
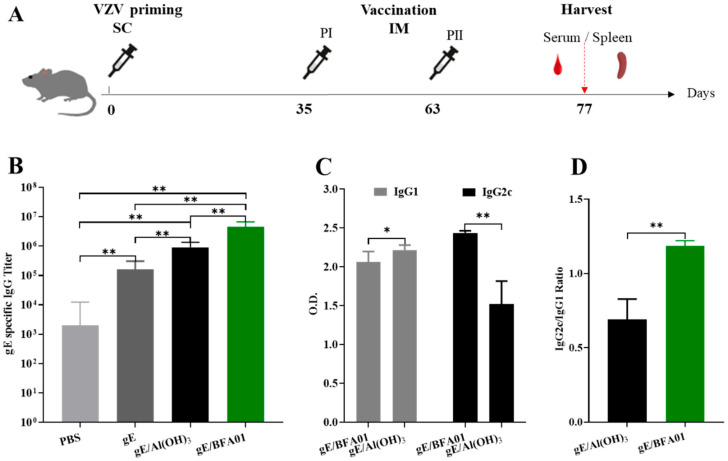
Immunization schedule and gE-specific IgG titers. (**A**) Schematic diagram of the vaccine immunogenicity evaluation experiment in C57BL/6N mice. Mice (*n* = 6 per group) were immunized subcutaneously with live attenuated VZV vaccine on d0, then immunized intramuscularly twice with 5 μg of gE alone or 5 μg of gE formulated with Al(OH)_3_ and BFA01 adjuvant on d35 and d63; meanwhile, the mice injected twice with PBS served as a control group. The mice were sacrificed 14 days after the last immunization and serum or spleen was collected for analysis. (**B**) gE-specific IgG antibody titers were detected using ELISA; geometric means with 95% confidence intervals are shown. (**C**) gE-specific IgG1 and IgG2c antibodies in the gE/BFA01 group and gE/Al(OH)_3_ group. (**D**) IgG2c/IgG1 ratio in the gE/BFA01 and gE/Al(OH)_3_ groups. IgG1 and IgG2c antibody levels were measured in 5000-fold diluted serum and the values were determined from the absorbance values measured at 450 nm; data are shown as the mean and standard deviation (SD). The Mann–Whitney test was used for the statistical analysis; * *p* < 0.05, ** *p* < 0.01.

The C57BL/6N mice were randomly divided into 4 groups, with 6 mice in each group. All animals in each group were primed subcutaneously in the neck scruff with approximately 2 × 10^3^ plaque-forming units (pfu) of VZV live attenuated virus vaccine (Oka-strain, SHANGHAI RONGSHENG BIOTECH CO., LTD, Shanghai, China) 35 days prior to vaccination. The right hind limb gastrocnemius muscle was then immunized with 50 μL of PBS, gE antigen (5 μg/animal), gE antigen formulated with Alhydrogel adjuvant (gE/Al(OH)_3_, gE/5 μg + 50 μg/animal), or gE antigen formulated with BFA01 adjuvant (gE/BFA01, gE/5 μg + 50 μL/animal). Each group received two immunizations with a 28-day interval. The comparison experiment on immunogenicity with Shingrix used the same immunization dose and schedule, with 10 mice in each group. Fourteen days after the second immunization, blood was collected from the posterior orbital venous plexus of the mice, and the serum was separated. The serum was tested for gE-specific IgG antibodies and subtypes. At the same time, spleen cells were isolated from the spleen for intracellular cytokine detection. The mouse immunization and immunogenicity assessment schedule are shown in Figure 1A.

#### 2.2.2. Measurement of gE-Specific Antibody Titers

gE-specific antibody levels in immunized mouse serum samples were determined using an indirect ELISA. Briefly, the recombinant gE protein diluted in PBS was pre-coated onto ELISA plates at a final concentration of 3 μg/mL. After overnight incubation at 4 °C, unbound gE was removed by washing two times with PBST (PBS containing 0.05% Tween 20). The plate was blocked for 2.5 h at 37 °C with 200 μL/well blocking buffer (PBST containing 0.05% Proclin 300 and 1% Bovine Serum Albumin (BSA)). After removing the blocking buffer, twofold-diluted serum samples were added to the plate, incubated for 90 min at 37 °C, and then washed 3 times with PBST. Binding IgG, IgG1, and IgG2c were determined using HRP-conjugated goat-anti-mouse IgG (1:150,000, Jackson ImmunoResearch Laboratory Inc., West Grove, PA, USA), IgG1 (1:20,000, Abcam, Cambridge, UK), and IgG2c (1:20,000, Abcam, UK) for 45 min at 37 °C. Plates were washed and incubated with a solution of 3,3′,5,5′-tetramethylbenzidine (TMB substrate (Suzhou Yacoo Science Co., Ltd., Suzhou, China)). The enzymatic reaction was terminated with 0.2 M sulfuric acid, and the plates were read at 450 nm with a microplate reader (BioTek Instruments, Winooski, VT, USA). IgG titers was defined as end-point dilutions greater than 2.1 times the mean OD value of the blank control. The IgG1 and IgG2c antibody detection methods are the same as those for IgG, except that the enzyme-labeled antibody is HRP-labeled anti-mouse IgG1 or IgG2c antibody (Abcam, UK).

#### 2.2.3. Intracellular Cytokine Staining

Intracellular cytokine staining (ICS) and flow cytometry were used to detect the gE-specific CD4^+^ T and CD8^+^ T cells, which express IFN-γ and/or IL-2. Spleens were harvested from the mice, and a splenic lymphocyte suspension was obtained by sieve grinding, followed by red blood cell lysis with a red blood cell lysis buffer (BioLegend, San Diego, CA, USA). The cell concentration was adjusted to 1 × 10^6^ cell/mL; the spleen cells (1 mL/tube) were stimulated in vitro using a VZV-gE peptide pool (20-mer peptides with 12-amino acid overlap, synthesized by GenScript Biotech Co., Ltd., Nanjing, China) in the presence of anti-CD28, anti-CD49d antibodies, and Brefeldin A (both BioLegend), and then incubated overnight at 37 °C in a 5% CO_2_ incubator. For cell staining, the single-cell suspension was washed with PBS and Fixable Viability Stain 450 (BD Biosciences, Franklin Lakes, NJ, USA) was added. After 20 min of incubation at 4 °C in the dark, the cell suspensions were washed, then resuspended with TruStain TcX (Fc blocking reagent, BioLegend) and incubated for 10 min. A mixture of fluorochrome-conjugated antibodies (against CD3e, CD4, and CD8a) were added and incubated 20 min at 4 °C for cell surface staining. After the cell washing, fixation, and permeabilization, the cells were incubated with anti-IFN-γ-APC and anti-IL-2-BV605 (both BD Bioscience) for intracellular staining at 4 °C and 60 min. The cell suspensions were washed and resuspended in PBS, live cells were gated (FSC/SSC), and data from all cells in each sample was collected using a NovoCyte flow cytometer (Agilent, Santa Clara, CA, USA). After analysis using NovoExpress 1.5.6 software, data on the cell percentages of the total frequencies of CD4^+^ and CD8^+^ T cells expressing IL-2 and/or IFN-γ were output.

#### 2.2.4. Enzyme-Linked Immunospot Assay

The enzyme-linked immunosorbent spot (ELISpot) assay was used to detect the number of gE-specific IFN-γ-secreting cells in the mouse spleens. The ELISpot assay was performed according to the manufacturer’s instructions. Pre-coated plates were blocked with a medium containing 10% FBS at room temperature for 30 min. After removing the medium, splenocytes (2.5 × 10^5^ cells/well) from immunized mouse were added to the plate. The cells were then stimulated for 18 h with a gE glycoprotein peptide pool. An equal volume of DMSO served as the negative control, while phorbol 12-myristate 13-acetate (PMA)/ionomycin acted as the positive control. After cell removal, spots were developed according to the manufacturer’s protocol. The spots were read and quantified using a CTL ImmunoSpot S6 UNIV reader (Cellular Technology Ltd. Shaker Heights, OH, USA).

### 2.3. Recruitment of Innate Immune Cells at the Injection Site and Activation of Lymph Node DCs

#### 2.3.1. Mouse Immunization

C57BL/6N mice were randomly divided into 4 groups: gE formulated with BFA01, BFA01 adjuvant, gE, and saline groups, with 30 mice in each. Each mouse was administered 50 μL immune samples via the gastrocnemius muscles of both hind limbs. The muscle tissue from the injection sites of five mice from each group was collected at 3, 24, 48, 72, 96, and 168 h post-immunization for the innate immune cell phenotype analysis. The bilateral inguinal lymph nodes of mice were collected at 24 h after immunization for DC activation analysis.

#### 2.3.2. Analysis of the Phenotype of Innate Immune Cells in the Muscle at the Vaccination Site

Gastrocnemius muscle tissue at the injection site were cut into small pieces and placed in 3 mL digestion solution containing DNase I and liberase (both Roche, Basel, Switzerland) for 30 min at 37 °C. Digestion was stopped by adding 5 mL RPMI 1640 containing 10% FBS at 4 °C for 5 min. The digested muscle tissue was ground and filtered through a 100 μm cell strainer to obtain a single-cell suspension. The cell suspension was layered on a mouse lymphocyte separation medium (Dakewe Biotech Co., Ltd., Shenzhen, China) and centrifuged at 20 °C and 800× *g* for 25 min to obtain muscle lymphocytes. After washing and resuspending in PBS, the lymphocytes were stained with the following antibodies: TruStain FcX, Fixable Viability Dye, eFluor 450 anti-mouse Ly-6C, Ly-6G Monoclonal Antibody (1A8-Ly6g) Super Bright600 (both Invitrogen, Carlsbad, CA, USA), PE anti-mouse CD11c, PE/Cyanine7 anti-mouse/human CD11b, APC anti-mouse I-A/I-E (MHC II), and FITC Anti-Mouse F4/80 (both BioLegend). After 20 min of incubation at 4 °C and washing using PBS, the cells were resuspended, and data collection and processing were performed using a NovoCyte flow cytometer (Agilent, Santa Clara, CA, USA). Neutrophils were defined as CD11b^+^ Ly6G^+^ cells, monocytes were defined as CD11b^+^ Ly6G^−^ Ly6C^+^ cells, macrophages were defined as CD11b^+^ F4/80^+^ cells, and dendritic cells (DCs) were defined as CD11c^+^ MHC II^+^ cells.

#### 2.3.3. Detection of DC Activation in Inguinal Lymph Nodes

Inguinal lymph nodes from both sides of the immunized mice were harvested, ground, and filtered through a 40 μm cell strainer. After centrifugation at 4 °C and 450× *g* for 10 min, the cells pellet was resuspended in PBS to prepare a single-cell suspension. 1 × 10^6^ cells were stained with the following antibodies: TruStain FcX, Fixable Viability Dye, PE anti-mouse CD11c, eFluor 450 anti-mouse CD40, Percp-eFluor 710 anti-mouse CD80, APC/Fire750 anti-mouse CD86, and APC anti-mouse I-A/I-E (MHC II). After centrifugation and resuspension in PBS, the cells were analyzed using a NovoCyte flow cytometer (Agilent, Santa Clara, CA, USA) to determine the proportions of CD40^+^ CD11c^+^, CD86^+^ CD11c^+^, CD80^+^ CD11c^+^, and MHC II^+^ CD11c^+^ cells.

### 2.4. Transcriptome Analysis of Inguinal Lymph Nodes After BFA01 Adjuvant Vaccination

#### 2.4.1. Mouse Immunization

The C57BL/6N mice were randomly divided into four groups, with three mice in each group. One group consisting of blank mice served as the control group, while the other three groups were immunized with 50 μL of BFA01 adjuvant via the bilateral hindlimb gastrocnemius muscle. Bilateral inguinal lymph nodes were collected 3, 6, and 24 h after immunization, and total RNA was extracted from the lymph nodes for transcriptomic analysis.

#### 2.4.2. Transcriptome Sequencing Analysis of Inguinal Lymph Nodes

Total RNA extracted from inguinal lymph nodes of mice in the control group (non-immunized) and 3, 6 and 24 h after immunization groups. mRNA was enriched using Oligo dT magnetic beads, then amplification and library construction were performed. Qualified libraries were sequenced using Illumina sequencing, and the high-quality data obtained were aligned with the reference genome to quantify gene expression levels. Differential expression genes between the two groups were screened using DESeq, and a KEGG pathway enrichment analysis was performed on the differentially expressed genes using ClusterProfiler software (3.8.1). Transcriptome analysis was performed by Novogene Co., Ltd. (Beijing, China).

#### 2.4.3. RT-qPCR Analysis of Cytokine Levels

Total RNA was extracted from lymph nodes of non-immunized mice or mice immunized with PBS or BFA01 adjuvant at 6 h post-immunization, using the RNA Easy Fast Tissue/Cell Kit (TIANGEN, Beijing, China) according to the manufacturer’s protocol. After quantifying the RNA with MicroDrop (BIO-DL, Shanghai, China), 2.5 μg of total RNA was used to obtain cDNA with the SMART MMLV Reverse Transcriptase Kit (Takara Bio USA, Inc, San Jose, CA, USA). RT-qPCR assays were performed using TB Green Fast qPCR Mix (Takara Bio Inc, Kusatsu, Shiga, Japan) according to the manufacturer’s instructions. qPCR was run on an Applied Biosystems 7500 Real-Time PCR system (Thermo Fisher Scientific, Waltham, MA, USA). Expression levels of target genes were normalized to HPRT1 [39], where the results were expressed as the fold change relative to the non-immune control group, corresponding to 2^−ΔΔCt^. The RT-qPCR primers are summarized in Supplementary Table S1.

### 2.5. Statistical Analysis

Statistical analyses were performed with GraphPad Prism 8.0.0 software. The serum gE antigen-specific antibody titers and frequencies of specific T cells are expressed as geometric means with 95% confidence intervals, while other results are expressed as the mean and standard deviation (SD). Intergroup comparisons were performed using the Mann–Whitney test. Differences were considered statistically significant when *p* < 0.05.

## 3. Results

### 3.1. Humoral Immune Response Induced by Recombinant Zoster Vaccine

Based on the pre-sensitization with the varicella live attenuated virus vaccine, the data of the serum gE-specific IgG antibodies and subtypes induced in the C57BL/6N mice on the 14th day after the second immunization are shown in Figure 1B–D. The geometric mean titer (GMT) of the serum IgG antibodies in the recombinant zoster vaccine (gE/BFA01) group was 4,525,483, which was 4.0 times that of the gE/Al(OH)_3_ group and 27.3 times that of the gE group (*p*-values were both 0.0022). The BFA01 adjuvant significantly enhanced the levels of gE-specific antibodies. Due to the presence of live attenuated varicella vaccine immunity, the IgG antibody GMT in the PBS group was 2000, which was significantly lower than those in the other three groups (Figure 1B).

Analysis results of the serum IgG antibody subtypes in the gE formulated with BFA01 and Al(OH)_3_ are shown in Figure 1C. The IgG1 antibody induced by the gE/Al(OH)_3_ group was significantly higher than that of the vaccine (gE/BFA01) group (*p* = 0.0152), while the IgG2c antibody was significantly lower than that in the gE/BFA01 group (*p* = 0.0022). The ratio of IgG2c to IgG1 in the gE/BFA01 group was 1.185, significantly higher than that in the gE/Al(OH)_3_ group (ratio of 0.689) (Figure 1D). The results indicate that compared with gE immunization alone, the Al(OH)_3_ and BFA01 adjuvants significantly enhanced the humoral immune response, and the immune response induced by BFA01 was biased toward the Th1 type.

### 3.2. Cell-Mediated Immune Response Induced by Recombinant Zoster Vaccine

**Figure 2:**
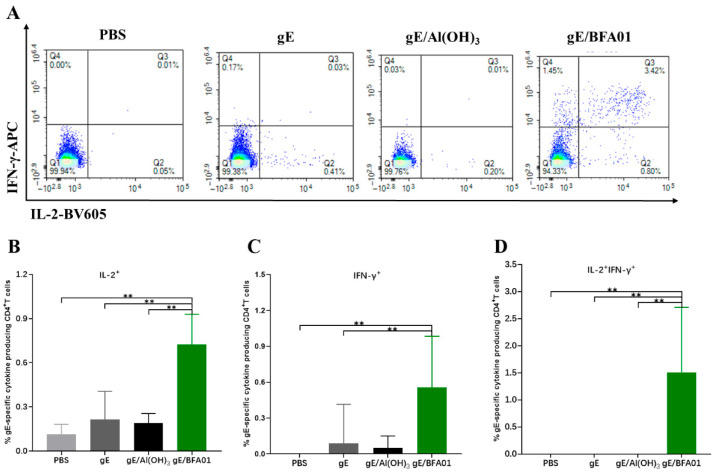
gE-specific CD4^+^ T cell responses induced by gE/BFA01 in C57BL/6N mice. Mice (*n* = 6 per group) were immunized subcutaneously with live attenuated VZV vaccine, then boosted two times with a 28-day interval. Spleens were collected and processed to obtain splenocytes at 14 days after the last immunization. Splenocytes were stimulated with a pool of peptides spanning the gE antigen (1.25 μg/mL) for 18 h. (**A**) Pseudocolor images displaying representative results for frequencies of CD4^+^ T cells expressing IFN-γ and/or IL-2. Frequencies of IL-2^+^ (**B**), IFN-γ^+^ (**C**), and IL-2^+^IFN-γ^+^ (**D**) CD4^+^ T cells were determined using flow cytometry; geometric means with 95% confidence intervals are shown. The Mann–Whitney test was used for the statistical analysis; ** *p* < 0.01.

The results of the gE-specific CD4^+^ T cell-mediated immune (CMI) response are shown in Figure 2. The proportions of IL-2^+^CD4^+^ T and IFNγ^+^CD4^+^ T cells induced by the vaccine (gE/BFA01) group were 3.4-fold (*p* < 0.01) and 5.1-fold (*p* < 0.01) higher than those in the gE group, respectively, and 3.8-fold (*p* < 0.01) and 8.7-fold higher than those in the gE/Al(OH)_3_ group, respectively (Figure 2B,C). The proportion of multifunctional T cells (IL-2^+^IFNγ^+^CD4^+^ T cells) induced by the gE/BFA01 group was significantly higher than those in the PBS, gE, and gE/Al(OH)_3_ groups (Figure 2D). These results indicate that the BFA01 adjuvant significantly enhances gE-specific cellular immunity.

### 3.3. Comparison of Immunogenicities of the gE/BFA01 and Shingrix Vaccines

**Figure 3:**
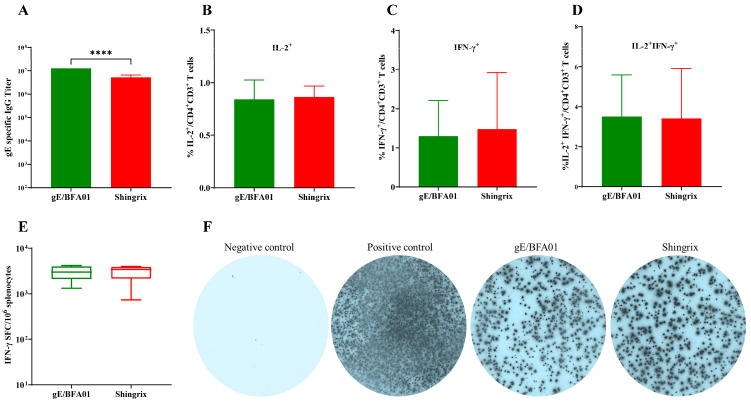
The gE/BFA01 vaccine induced humoral and cellular immune responses at levels comparable with Shingrix in C57BL/6N mice. Mice (*n* = 10 per group) were immunized subcutaneously with a live attenuated VZV vaccine, then immunized intramuscularly twice with 5 μg of gE/BFA01 or Shingrix vaccine with a 28-day interval. Serum and spleen were collected at 14 days after the last immunization for analysis. (**A**) gE-specific IgG antibody titer measured using ELISA. (**B**–**D**) Splenocytes were stimulated with a pool of peptides spanning the gE antigen (1.25 μg/mL) for 18 h. Frequencies of IL-2^+^ (**B**), IFN-γ^+^ (**C**), and IL-2^+^IFN-γ^+^ (**D**) CD4^+^ T cells were determined using ICS. (**E**) The expression of IFN-γ from isolated splenocytes after stimulation with gE peptides were measured using ELISPOT. (**F**) Representative images of IFN-γ-producing splenocytes in the ELISpot assay. Data are shown as geometric means with 95% confidence intervals. The Mann–Whitney test was used for statistical analysis; **** *p* < 0.0001.

Shingrix has been approved by the U.S. FDA for herpes zoster prevention in the elderly population, demonstrating 97.2% efficacy in individuals aged 50 years and older. To further evaluate the immune response to gE/BFA01, we compared the humoral and cellular immune responses between gE/BFA01 and Shingrix (Figure 3). We found that the gE/BFA01 induced significantly higher IgG antibody titers than Shingrix (*p* < 0.0001, Figure 3A). The ICS results show no significant differences in the CMI responses (IL-2^+^CD4^+^ T cells, IFN-γ^+^CD4^+^ T cells, IL-2^+^IFN-γ^+^CD4^+^ T cells) induced by gE/BFA01 and Shingrix (Figure 3B–D). ELISpot results further confirmed no significant differences in IFN-γ-secreting cells induced by the two vaccines (Figure 3E,F). These findings indicate that the gE/BFA01-induced CMI response is comparable with that of Shingrix. Based on the research on the pathogenesis of HZ, cellular immunity plays the critical role in preventing herpes zoster rather than antibody levels. We will further compare the immunogenicity of the gE/BFA01 and Shingrix vaccines in clinical trials.

### 3.4. Innate Immune Cell Recruitment Effect of BFA01 Adjuvant

**Figure 4:**
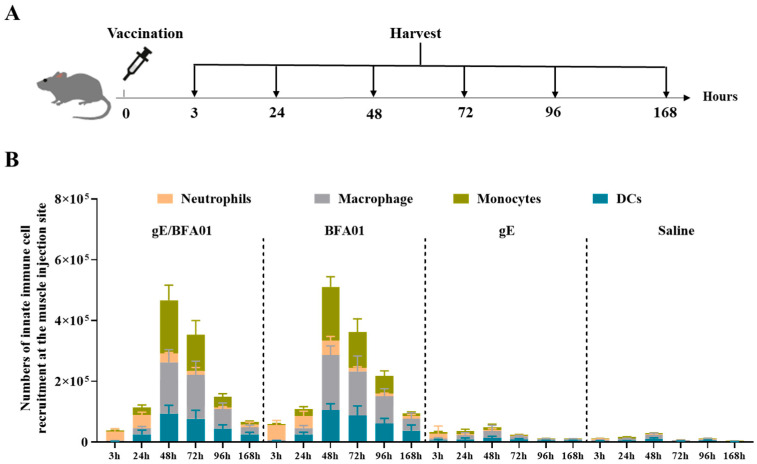
The BFA01 adjuvant enhanced the number of innate immune cells at the injection site of the C57BL/6N mice. (**A**) Schematic diagram of mouse experiments. The mice were immunized with a single dose of BFA01 or/and gE antigen via the gastrocnemius muscles of both hind limbs; the injection site muscles were isolated for 3–168 h (=day 7) post-injection and analyzed using flow cytometry. The mice injected with saline served as a control group. (**B**) Number of innate immune cells in the muscle injection site at different timepoints; data are shown as mean + SD (*n* = 5).

Following enzymatic digestion of the muscle injection site, cell phenotyping was performed via flow cytometry to evaluate innate immune cell recruitment. The mouse immunization and sampling schedule is shown in Figure 4A. Changes in the innate immune cell populations over 7 days post-immunization were monitored using the gating strategy depicted in Supplementary Figure S2.

Figure 4B shows that the recombinant zoster vaccine (gE/BFA01 group) exhibited consistent responses with BFA01 adjuvant, and various types of innate immune cells could be recruited 3–168 h after intramuscular administration. Neutrophils (CD11b^+^ Ly6G^+^) appeared earliest and reached a peak at 3–24 h, followed by a rapid decline. After 24 h, monocytes (CD11b^+^ Ly6G^−^ Ly6C^+^), macrophages (CD11b^+^ F4/80^+^), and dendritic cells (CD11c^+^ MHC II^+^) rapidly increased, peaking at 48 h post-immunization and declining to lower levels by 168 h (day 7). When the gE was administered alone, only minimal numbers of innate immune cells were observed, suggesting that the immune cell recruitment at the injection site in the vaccine (gE/BFA01) group was entirely driven by the BFA01 adjuvant, while the gE antigen provided only a minimal contribution.

### 3.5. The BFA01 Adjuvant Induced DC Activation

**Figure 5:**
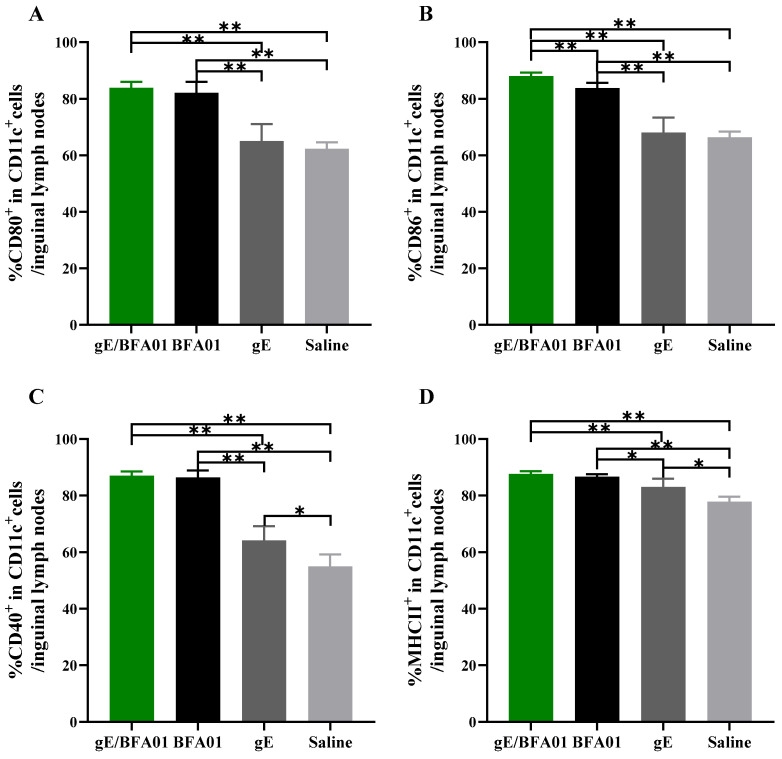
The BFA01 adjuvant activated DCs in the inguinal lymph nodes of C57BL/6N mice. Mice were immunized with a single dose of BFA01 or/and gE antigen via gastrocnemius muscles of both hind limbs, and the inguinal lymph nodes were collected 24 h after immunization and processed to obtain the cell suspension. Flow cytometry was performed to measure the expressions of CD80 (**A**), CD86 (**B**), CD40 (**C**), and MHC II (**D**) in live DCs (FVD506^−^CD11c^+^ cells) from LN; data are shown as mean + SD (*n* = 5). The Mann–Whitney test was used for statistical analysis; * *p* < 0.05, ** *p* < 0.01.

Dendritic cells (DCs) are the most effective professional antigen-presenting cells (APCs). Co-stimulatory molecules, such as CD80 and CD86, provide the essential second signal during antigen peptide presentation from dendritic cells to T cells [40]. The expression of co-stimulatory molecules typically depends on signals from inflammatory cytokines (e.g., TNFα) and pathogen-associated molecular patterns (PAMPs). To assess the role of the BFA01 adjuvant and vaccine in inducing an immune response, inguinal lymph nodes were collected 24 h after vaccination with the BFA01 adjuvant and vaccine, and the expression levels of co-stimulatory molecules on DCs were analyzed (Figure 5) to evaluate their activation status. Compared with the saline group, gE alone did not enhance the expression of CD80 and CD86 co-stimulatory molecules, and CD40 and MHC II markers were only marginally higher than in the saline group. However, the BFA01 adjuvant group significantly outperformed the saline and gE groups in inducing expression of all four maturation markers. Meanwhile, BFA01’s ability to induce co-stimulatory molecule expression was comparable with the vaccine group (excluding CD86, where gE/BFA01 was higher than BFA01 alone). These findings indicate that the gE antigen alone cannot effectively induce co-stimulatory molecule expression in BMDCs; instead, it requires combination with BFA01 adjuvant to enhance the immune response.

### 3.6. Changes in Gene Expression in Lymph Nodes Induced by BFA01 Adjuvant

**Figure 6:**
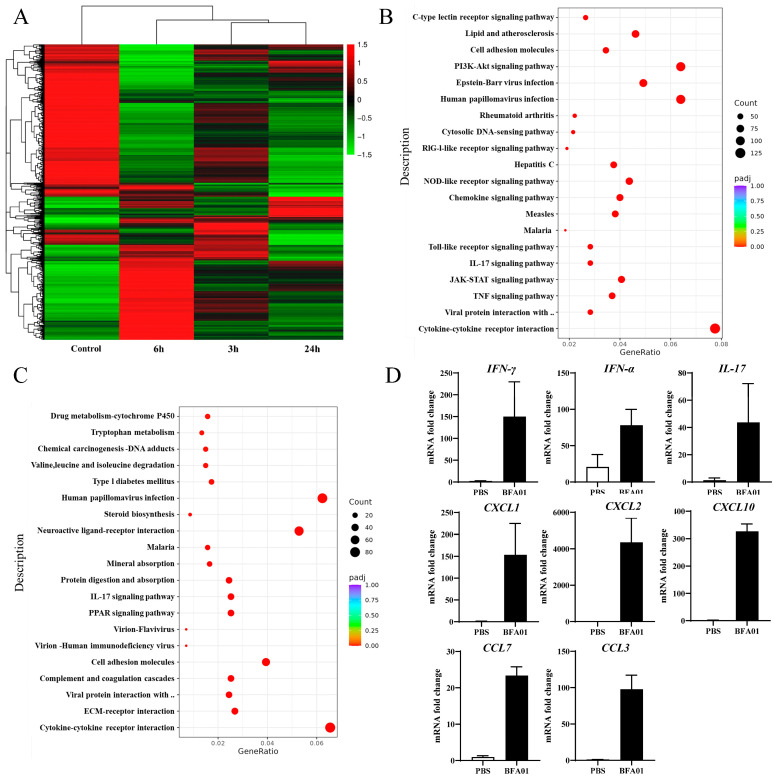
Modulation of gene expression after BFA01 adjuvant immunization in C57BL/6N mice. Mice were intramuscularly immunized once with BFA01 adjuvant or PBS, while non-immunized mice served as the control. The inguinal lymph nodes were collected at the indicated time points for analysis. (**A**) Heatmap of differentially expressed genes in inguinal lymph nodes at 3, 6, and 24 h after the BFA01 immunization. (**B**) Scatter plot of the enrichment analysis for differentially expressed genes at 6 h (the *x*-axis represents the ratio of the number of differentially expressed genes annotated to KEGG pathways to the total number of differentially expressed genes, and the *y*-axis represents the KEGG pathways; the same applies below). (**C**) Scatter plot of the enrichment analysis for differentially expressed genes at 24 h. (**D**) Cytokine and chemokine gene levels in dLN at 6 h after treatment. Data are shown as mean + SD (*n* = 3).

The heatmap and differential expressed genes analysis in the inguinal lymph nodes of BFA01 adjuvant-immunized C57BL/6N mice at 3, 6, and 24 h post-immunization showed that gene expression in the lymph nodes underwent significant changes as early as 3 h post-immunization, with the highest number of differentially expressed genes and the greatest magnitude of change at 6 h. At 24 h, the number of differentially expressed genes slightly decreased, and the number of downregulated differentially expressed genes significantly increased (Figure 6A).

The KEGG (Kyoto Encyclopedia of Genes and Genomes) pathway enrichment analysis results show that the differentially expressed genes at 3 and 6 h primarily participated in the TNF, IL-17, TLR, and JAK-STAT signaling pathways (Figure 6B,C). TNF and TLR signals promote the activation of immune cells, such as macrophages and dendritic cells, during innate immune responses [41,42]. The TNF signaling pathway modulates immune responses by secreting other cytokines (e.g., IL-1, IL-6) and participates in T cell differentiation and survival, as well as the regulation of adhesion molecules like ICAM-1 and selectin-E in endothelial cells [43]. At 6 h, molecules associated with the PI3K-AKT signaling pathway were significantly upregulated. The PI3K-AKT signaling pathway is crucial for cell survival and cell cycle initiation. At 24 h, the ECM–receptor interaction and cell adhesion molecules unrelated to T cell activation (except CD80/PD-L1) were downregulated, while the expression of molecules associated the TNF and TLR signaling pathways approached baseline levels, indicating that the innate immune response had been downregulated and the adaptive immune response had been initiated. The transcriptional expression analysis of a set of cytokines via qPCR confirmed the differentially expressed genes identified in the transcriptome analysis (Figure 6D).

## 4. Discussion

VZV antibodies provide protection against varicella caused by primary VZV infection, but their role in preventing reactivation of latent infection, preventing HZ, and reducing the severity of PHN is limited [44]. In contrast, the T-cell-mediated immunity (CMI) plays a key role in controlling viral replication during VZV reactivation [45]. In the control of VZV reactivation, CD4^+^ T cells capable of secreting Th1-type cytokines such as IFN-γ and IL-2 are particularly important, and a decrease in their numbers is associated with an increase in the incidence of herpes zoster [46]. In this study, two doses of the recombinant herpes zoster vaccine based on the BFA01 adjuvant were administered in a VZV-primed animal model, inducing high levels of IL-2^+^, IFN-γ^+^, and IL-2^+^IFN-γ^+^ CD4^+^ T cell responses (Figure 2). The results of this study also confirm that compared with the Al(OH)_3_ adjuvant, which is difficult to effectively induce cellular immune responses [47], the BFA01 adjuvant shows significant advantages in enhancing humoral and cellular immune responses induced by recombinant gE. They can induce Th1-biased immune responses, especially with a significant increase in the proportion of IFN-γ and IL-2 double-positive multifunctional CD4^+^ T cells (Figure 1C and Figure 2).

In the field of herpes zoster prevention, the recombinant VZV gE antigen combined with the lipid-based adjuvant AS01_B_ recombinant protein vaccine (Shingrix) holds a leading position in the global market [48]. Beyond Shingrix, multiple novel adjuvant-containing recombinant shingles vaccines are currently in development, incorporating various immunostimulants, such as CpG1018, CpG2006, poly(I:C), PLGA, dLOS, and aluminum hydroxide [35,36,49,50,51,52,53,54]. Among these, the cationic liposome adjuvant CIA09 based on TLR4 agonist dLOS and QS-21 significantly increases the proportion of CD4^+^ T cells expressing IFN-γ, TNFα, and IL-2 [54]. In a direct comparative study between LZ901 (an aluminum hydroxide adjuvant-based vaccine) and Shingrix, LZ901 induced comparable antibody levels but higher cellular immune responses [49]. In our study, we compared the gE/BFA01 vaccine with Shingrix, the most effective vaccine against herpes zoster. The adjuvant activity of AS01_B_ depends on the synergistic effects of MPL and QS-21 to enhance the secretion of Th1 type of cytokines, such as IL-2, IFN-γ, and IgG2a; circulation of granulocytes; and recruitment of monocytes and dendritic cells, subsequently strengthening the humoral and antigen-specific Th1-biased cellular immune responses [55]. The comparative analysis showed that the gE/BFA01 vaccine induced cellular immune responses comparable to Shingrix. The gE/BFA01 vaccine group exhibited higher IgG antibody titers, but there was no evidence linking antibody levels to protective efficacy. Although our adjuvant BFA01 shares the same immunostimulants MPL and QS-21 with AS01_B_, the adjuvant formulations differ, and MPL and QS-21 are manufactured by separate entities. Consequently, concentrations of key active ingredients may vary, potentially contributing to higher antibody titers [56,57]. However, these differences do not alter the fundamental TLR4 agonist properties of the MPL adjuvant, enabling it to induce comparable CD4^+^ T cell responses [58].

In the body’s anti-infective immune response, neutrophils can rapidly control local inflammation and participate in antigen clearance, while monocytes differentiate locally into macrophages to participate in eliminating inflammatory responses, processing antigens, and assisting DCs in stimulating CD4^+^ T cell differentiation [59]. DCs, as professional antigen-presenting cells, mature after antigen uptake, upregulate the expression of MHC-II and co-stimulatory molecules (CD80, CD86, etc.), and effectively activate T cells and B cells, thereby initiating adaptive immune responses and immune memory. In the early stages of the immune response, rapid activation of DCs is a key factor in triggering T cell responses. Activation of DCs can directly improve the T cell activation efficiency [60,61]. This study demonstrates that the BFA01 adjuvant transiently recruits many innate immune cells at the injection site (muscle), leading to rapid activation of the innate immune response at the injection site. Consistent with previous reports [62], neutrophils are the first to respond, followed by a rapid increase in monocytes within 24 h, which are one of the most abundantly recruited innate immune cell populations. Dendritic cells continue to increase after injection and reach a peak at 48 h. This contrasts with the findings of Arnaud et al., where DC counts showed no significant change over 7 days, likely because no additional time points were set between 16 and 168 h [62]. The BFA01 adjuvant and the recombinant zoster vaccine based on BFA01 rapidly recruit innate immune cells and activate DCs, promoting and enhancing immune responses, which plays a crucial role in improving age-related cellular immune compromise.

By analyzing the signaling pathways involved in the differentially expressed genes at different immune time points, the significant enrichment of the TLR signaling pathway at 3 and 6 h may be related to the MPL, which acts as a TLR4 agonist. The TNF signaling pathway was also significantly upregulated at 3 and 6 h, which is speculated to be related to the stimulation of TNF-α secretion by macrophages in lymph nodes. Sophie et al. demonstrated that CD169+ resident macrophages in dLN play a central role in the adjuvanticity of QS-21 [63]. Margherita et al. observed significant the enrichment of interferon-related signals in a study of the AS01 adjuvant mechanism [39], differing from our findings where no interferon-related signaling pathways were identified. However, we also observed significantly elevated expression levels of IFN-γ and IFN-α (Figure 6D). These discrepancies may stem from the use of different databases for analysis. An intriguing finding in KEGG pathway analysis was a significant enrichment of cytosolic DNA-sensing pathway and RIG-I-like receptor signaling pathway, with ACOD1 emerging as the most highly upregulated differentially expressed gene. O’Carroll et al. found that LPS induces ACOD1 upregulation, converting succinic acid to itaconate in the tricarboxylic acid cycle. Itaconic acid then inhibits succinate dehydrogenase, leading to the release of double-stranded mitochondrial RNA [64]. Paulenda et al. further demonstrated that itaconate induces mitochondrial oxidative stress by inhibiting PRDX5 activity, disrupting mitochondrial membrane integrity and releasing mitochondrial DNA [65,66]. Both studies confirm that LPS induces the release of mitochondrial DNA and RNA. Therefore, it can be inferred that the cytosolic DNA-sensing pathway and RIG-I-like receptor signaling pathway observed in our results are associated with MPL.

The limitation of this study was that all experiments were conducted in mice. Although the VZV pre-sensitization model was used to simulate the seropositive status in adults, since mice are not the natural host of VZV and cannot develop true latent infection and reactivation processes, it is impossible to evaluate the actual protective efficacy of the vaccine against herpes zoster onset. Therefore, the efficacy can only be indirectly inferred through immunological indicators.

## 5. Conclusions

Aging and impaired immune function are associated with an increased risk of developing herpes zoster and its complications. Enhancing cell-mediated immune responses is key to controlling the reactivation of latent varicella-zoster virus. A series of experiments conducted on C57BL/6N mice demonstrated that the self-developed recombinant herpes zoster vaccine gE/BFA01 can induce strong humoral and cellular immune responses targeting the gE antigen. The BFA01 adjuvant induces innate immune responses and adaptive immune responses by recruiting innate immune cells, activating antigen-presenting cells, and modulating related signaling pathways.

## Data Availability

The original contributions presented in this study are included in the article/Supplementary Materials. Further inquiries can be directed to the corresponding authors.

## Supplementary Materials

The following supporting information can be downloaded at: https://www.mdpi.com/article/10.3390/v18010053/s1. Table S1: Overview of qPCR primers. Figure S1: Results of detection for purified gE protein and BFA01 adjuvant. Figure S2: Representative gating strategy for flow cytometry analysis of innate immune cell in the muscle injection site of mice.
