# Supplementary material for: Immunogenicity of a Recombinant Zoster Vaccine (gE/BFA01) in Mice
Source: Viruses. 2025 Dec 30;18(1):53. doi: 10.3390/v18010053 (PMC12846642; doi:10.3390/v18010053)
Supplement: Supplementary file 1 [file viruses-18-00053-s001.zip › viruses-4027132-supplementary.pdf]

Supplementary Materials

Table S1 Overview of qPCR primers

Gene	Forward Primer	Reverse Primer
CXCL1	TGCACCCAAACCGAAGTCAT	ACTTGGGGACACCTTTTAGCA
CXCL2	GAAGACCCTGCCAAGGGTTG	AGGCAAACTTTTTGACCGCC
CXCL3	TGTTGTGGCCAGTGAGCTG	ACTTCTGTCTGGGTGCAGTG
CXCL10	CCAAGTGCTGCCGTCATTTT	CTCAACACGTGGGCAGGATA
CCL2	CACTCACCTGCTGCTACTCA	GGTCAGCACAGACCTCTCTC
CCL3	CCCAGCCAGGTGTCATTTTCCT	CAGGCATTCAGTTCCAGGTCA
CCL4	CTCTCTCTCCTCTTGCTCGTG	CTGCCGGGAGGTGTAAGAGA
CCL7	GATCTCTGCCACGCTTCTGT	ATAGCCTCCTCGACCCACTT
IL-17	ACTACCTCAACCGTTCCACG	TTCCCTCCGCATTGACACAG
IL-18	GGCTGCCATGTCAGAAGACT	GTCTGGTCTGGGGTTCACTG
IFN-α	AATTTCCCCTGACCCAGGAAGATG	GTCTTCCCAGCACATTGGCAG
IFN-γ	GCATTCATGAGTATTGCCAAGTTTG	CGGATGAGCTCATTGAATGCTT
HPRT1	GCAAACTTTGCTTTCCCTGG	ACTTCGAGAGGTCCTTTTCACC

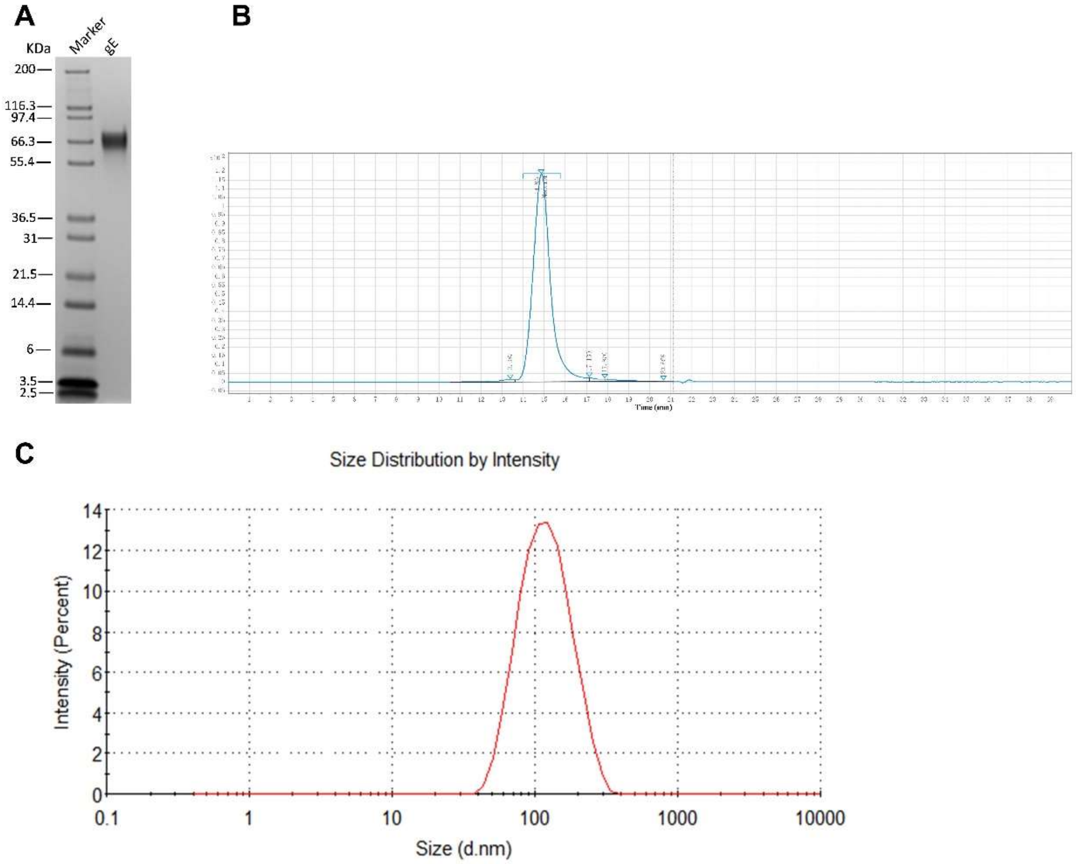

Supplementary Figure S1 Results of detection for purified gE protein and BFA01 adjuvant: (A): SDS-PAGE analysis of recombinant gE protein. (B): The purity of recombinant gE protein was

measured by SEC-HPLC. (C): Measurements of the BFA01 particle size.

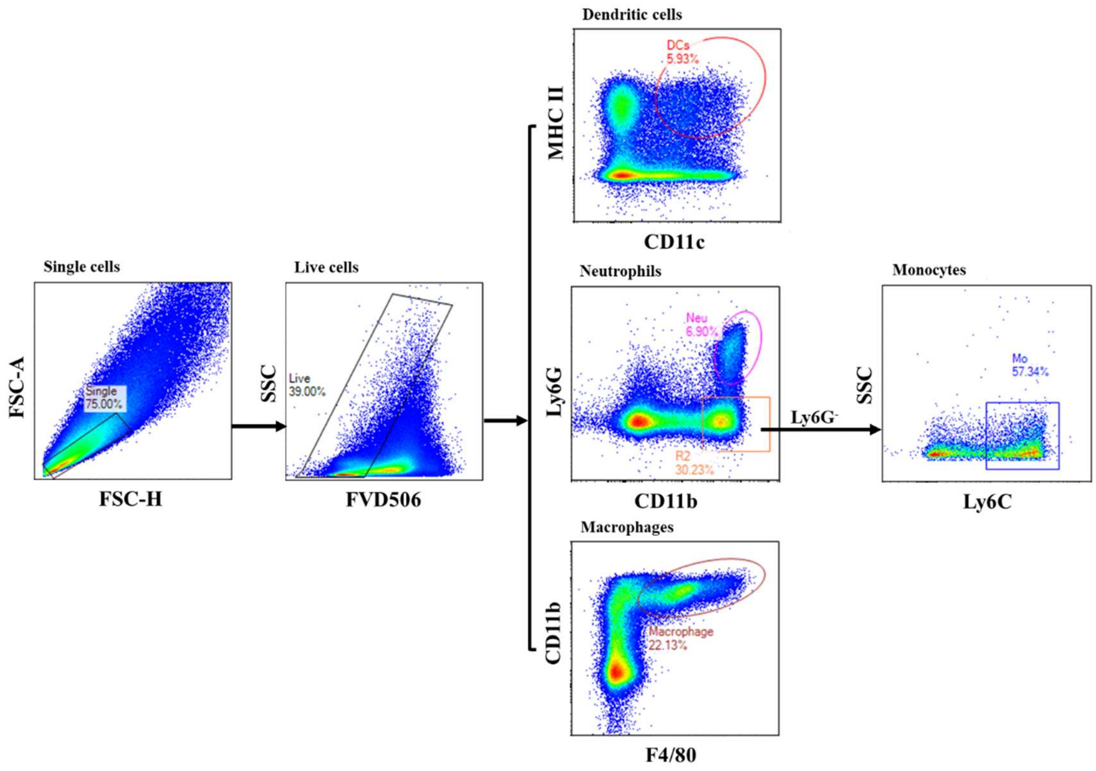

Supplementary Figure S2 Representative gating strategy for flow cytometry analysis of innate immune cell in the muscle injection site of mice. Firstly, we identified the single cells with FSC-H/FSC-A and live cells with Fixable Viability Dye (FVD605) markers in the lymphocytes isolated from the muscle of injection site of immunized mice. From $FDV605^-$ population, we gated over CD11c and MHC II and identified dendritic cells ($CD11c^+$ MHC II^+), gated over CD11b and Ly6G and identified neutrophils ($CD11b^+$ $Ly6G^+$), and from the $CD11b^+Ly6G^-$ population, we identified monocytes ($CD11b^+$ $Ly6G^-$ $Ly6C^+$) based on the Ly6C surface markers, and gated over CD11b and F4/80 and identified macrophages ($CD11b^+$ $F4/80^+$).
